# Cytokine-mediated changes in K^+^ channel activity promotes an adaptive Ca^2+^ response that sustains β-cell insulin secretion during inflammation

**DOI:** 10.1038/s41598-018-19600-x

**Published:** 2018-01-18

**Authors:** Matthew T. Dickerson, Avery M. Bogart, Molly K. Altman, Sarah C. Milian, Kelli L. Jordan, Prasanna K. Dadi, David A. Jacobson

**Affiliations:** 1 Department of Molecular Physiology and Biophysics, Vanderbilt University, Nashville, TN USA; 2 Department of Biological Sciences, Ohio University, Athens, OH USA

## Abstract

Cytokines present during low-grade inflammation contribute to β-cell dysfunction and diabetes. Cytokine signaling disrupts β-cell glucose-stimulated Ca^2+^ influx (GSCI) and endoplasmic reticulum (ER) Ca^2+^ ([Ca^2+^]_ER_) handling, leading to diminished glucose-stimulated insulin secretion (GSIS). However, cytokine-mediated changes in ion channel activity that alter β-cell Ca^2+^ handling remain unknown. Here we investigated the role of K^+^ currents in cytokine-mediated β-cell dysfunction. K_slow_ currents, which control the termination of intracellular Ca^2+^ ([Ca^2+^]_i_) oscillations, were reduced following cytokine exposure. As a consequence, [Ca^2+^]_i_ and electrical oscillations were accelerated. Cytokine exposure also increased basal islet [Ca^2+^]_i_ and decreased GSCI. The effect of cytokines on TALK-1 K^+^ currents were also examined as TALK-1 mediates K_slow_ by facilitating [Ca^2+^]_ER_ release. Cytokine exposure decreased *KCNK16* transcript abundance and associated TALK-1 protein expression, increasing [Ca^2+^]_ER_ storage while maintaining 2^nd^ phase GSCI and GSIS. This adaptive Ca^2+^ response was absent in TALK-1 KO islets, which exhibited decreased 2^nd^ phase GSCI and diminished GSIS. These findings suggest that K_slow_ and TALK-1 currents play important roles in altered β-cell Ca^2+^ handling and electrical activity during low-grade inflammation. These results also reveal that a cytokine-mediated reduction in TALK-1 serves an acute protective role in β-cells by facilitating increased Ca^2+^ content to maintain GSIS.

## Introduction

Failure of β-cells to secrete sufficient insulin precedes the onset of type 2 diabetes mellitus (T2DM)^1^. As the incidence of T2DM is rapidly increasing, it is important to identify better therapeutic options for reducing β-cell failure during the pathogenesis of the disease. Low-grade inflammation is a key contributor to β-cell dysfunction in T2DM^1–8^. Conditions of over-nutrition and inactivity result in low-grade systemic inflammation during which pro-inflammatory cytokine concentrations (e.g. tumor necrosis factor-α (TNF-α), interleukin-1β (IL-1β), and interferon-γ (IFN-γ)) increase several fold over basal levels^1–4,8–10^. For example, in a rat model of T2DM pancreatic cytokine levels were all elevated above nontreated controls (e.g. TNF-α increased from 24.3 ± 3.6 pg/mg protein to 47.9 ± 3.5 pg/mg protein (P < 0.05), IL-1β increased from 25.5 ± 2.7 pg/mg protein to 29.2 ± 1.7 pg/mg protein (P < 0.05), and IFN-γ increased from 49.4 ± 4.2 pg/mg protein to 65.1 ± 6.7 pg/mg protein (P < 0.05))^11^. The presence of these cytokines contributes to insulin resistance and diminished β-cell function^5^. Under stressful conditions (e.g. glucolipotoxicity) β-cells are also capable of secreting pro-inflammatory cytokines, which damage islet function^4,12^. Cytokine-mediated islet dysfunction correlates with increased basal intracellular Ca^2+^ ([Ca^2+^]_i_), reduced glucose-stimulated Ca^2+^ influx (GSCI), increased [Ca^2+^]_i_ oscillation frequency, altered endoplasmic reticulum (ER) Ca^2+^ ([Ca^2+^]_ER_) storage, and increased apoptotic signaling^5–7,13^. While chronic low-grade inflammation leads to β-cell dysfunction in T2DM, the mechanisms responsible remain unresolved. Understanding how cytokines disrupt islet Ca^2+^ handling may illuminate therapeutic targets for preventing β-cell failure during T2DM.

Calcium enters β-cells through voltage-dependent Ca^2+^ channels (VDCCs) that are controlled by ion channel-mediated changes in plasma membrane potential (*V*_m_)^14,15^. Thus, cytokine-mediated perturbations in Ca^2+^ handling would be predicted to result from changes in ion channel activity or expression. For example, accelerated islet [Ca^2+^]_i_ oscillations may be influenced by changes in K^+^ currents that control [Ca^2+^]_i_ oscillations such as Ca^2+^-activated K^+^ (K_Ca_), ATP-sensitive K^+^ (K_ATP_) channels, and/or the two-pore domain K^+^ (K2P) channel TALK-1^14,16,17^. K_slow_ is a K_Ca_ current controlled in part by [Ca^2+^]_ER_ release that senses and controls [Ca^2+^]_i_ by hyperpolarizing β-cell *V*_m_^16,18–22^. In β-cells it is believed to be composed of intermediate conductance (IK) K_Ca_, apamin-insensitive small conductance (SK) K_Ca_, and/or K_ATP_ channels^18,21,22^. TALK-1, while not sensitive to Ca^2+^, tunes islet [Ca^2+^]_i_ oscillation frequency, partly by facilitating [Ca^2+^]_ER_ release and activating K_slow_^14,23–27^. Interestingly, the transcript abundance of some of these K^+^ channels is decreased following cytokine exposure, including *KCNK16* (gene encoding TALK-1) and *ABCC8* (gene encoding sulfonylurea receptor 1 (SUR1))^28^. Likewise, mitochondrial function is reduced following cytokine exposure, which decreases ATP production and would be predicted to activate K_ATP_ channels^29^. This suggests that cytokine-induced changes in K^+^ channel function modulate [Ca^2+^]_i_ oscillation frequency during the pathogenesis of T2DM.

To further reveal how cytokines dysregulate β-cell [Ca^2+^]_i_ we investigated the electrophysiological mechanisms responsible for defective β-cell Ca^2+^ handling during low-grade inflammation. A cytokine-mediated increase in β-cell electrical oscillations was identified, which resulted from *V*_m_ depolarization and shorter interburst interval duration. Furthermore, these results demonstrated that K_slow_ and TALK-1 currents modify β-cell Ca^2+^ handling to preserve GSIS during acute low-grade inflammation representative of T2DM.

## Results

### Cytokine exposure decreases *KCNK16* transcript abundance and associated TALK-1 protein expression

**Figure 1:**
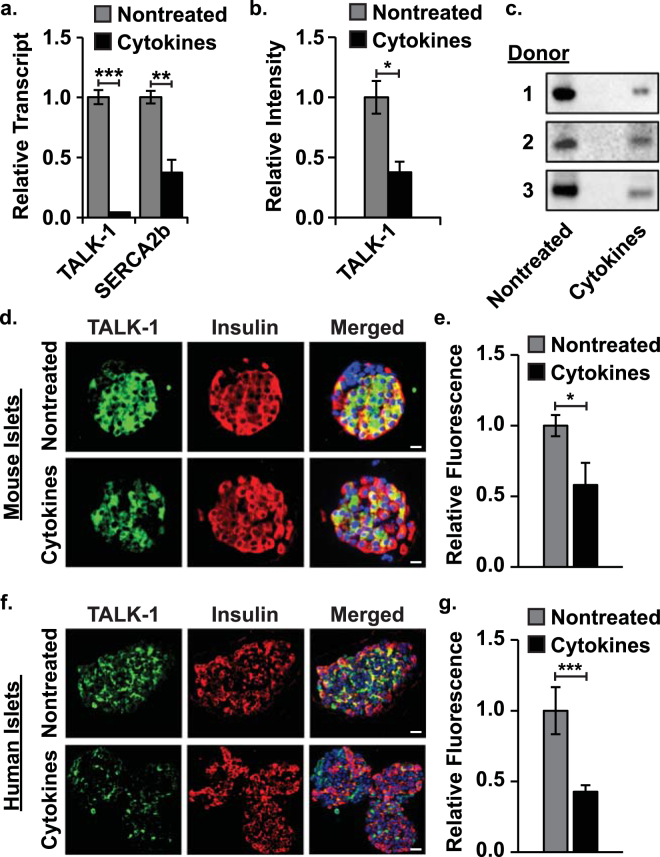
Cytokine exposure reduces islet *KCNK16* transcript abundance and associated TALK-1 protein. (**a**) qRT-PCR analysis of *KCNK16* (encodes TALK-1) and *ATP2A2* (encodes SERCA2b) transcript relative to *GAPDH* in nontreated (gray) and cytokine treated (black) WT mouse islets (N = 4 animals), (**b**) western blot analysis of human islet TALK-1 protein content with (black) and without (gray) cytokines (N = 3 donors), (**c**) images of human islet TALK-1 western blots for all donors, (**d**) representative immunofluorescent images of nontreated (upper panels) and cytokine treated (lower panels) mouse islet slices (TALK-1 - green, insulin - red, and nucleus - blue; scale bars are 20 µm), (**e**) average TALK-1 fluorescence intensity in nontreated (gray) and cytokine treated (black) mouse islet slices (N ≥ 3 islet slices), (**f**) representative immunofluorescent images of nontreated (upper panels) and cytokine treated (lower panels) human islet slices, and (**g**) average TALK-1 fluorescence intensity in nontreated (gray) and cytokine treated (black) human islet slices (N ≥ 5 islet slices). Statistical analysis was conducted using unpaired two-tailed t-tests and uncertainty is expressed as SEM (*P < 0.05, **P < 0.01, ***P < 0.001).

To investigate the effect of low-grade inflammation on islet TALK-1 transcript and protein expression, islets were treated for 24 hrs with a low concentration of cytokines. Quantitative RT-PCR revealed a loss of *KCNK16* (encodes TALK-1) and *ATP2A2* (encodes sarco/endoplasmic reticulum Ca^2+^-ATPase 2b (SERCA2b)) transcript in mouse islets following cytokine exposure (*KCNK16*: 95.63 ± 5.89% reduction and *ATP2A2*: 62.81 ± 11.95% reduction, Fig. 1a, P < 0.01). Western blotting of human islets also showed a cytokine-mediated reduction in TALK-1 protein (63.79 ± 14.81% reduction, Fig. 1b, c, P < 0.05). Similarly, cytokine exposure reduced TALK-1 immunofluorescence in mouse and human islet slices (mouse: 42.05 ± 17.33% reduction and human: 57.26 ± 17.25% reduction, Fig. 1d–g, P < 0.05).

### Cytokine exposure alters islet Ca^2+^ handling

**Figure 2:**
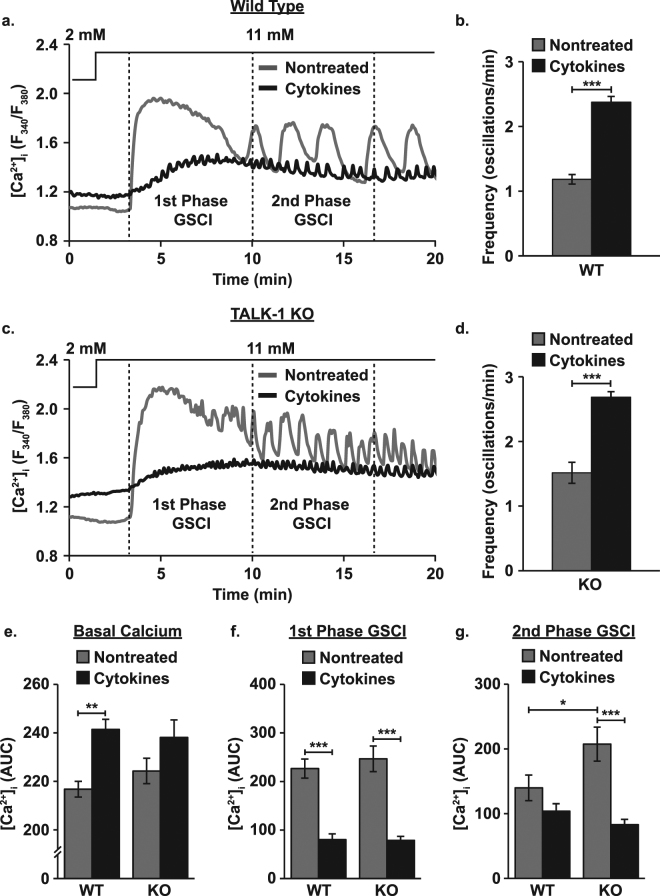
Cytokine exposure elevates basal islet [Ca^2+^]_i_ and disrupts GSCI. (**a**) Representative Fura-2 AM recordings (F_340_/F_380_) of changes in β-cell [Ca^2+^]_i_ for nontreated (gray) and cytokine treated (black) WT mouse islets (the lines above the figure indicate glucose concentrations), (**b**) average [Ca^2+^]_i_ oscillation frequency for nontreated (gray) and cytokine treated (black) WT mouse islets with 11 mM glucose (N ≥ 12 islets), (**c**) representative Fura-2 AM recordings of changes in β-cell [Ca^2+^]_i_ for nontreated (gray) and cytokine treated (black) TALK-1 KO mouse islets, (**d**) average [Ca^2+^]_i_ oscillation frequency for nontreated (gray) and cytokine treated (black) TALK-1 KO mouse islets with 11 mM glucose (N ≥ 30 islets), (**e**) average basal [Ca^2+^]_i_ AUC (0–200 sec) for nontreated (gray) and cytokine treated (black) WT and TALK-1 KO islets (N = 3 animals), (**f**) average 1^st^ phase GSCI AUC (200–600 sec) for nontreated (gray) and cytokine treated (black) WT and TALK-1 KO islets (N = 3 animals), and (**g**) average 2^nd^ phase GSCI AUC (600–1000 sec) for nontreated (gray) and cytokine treated (black) WT and TALK-1 KO islets (N = 3 animals). Statistical analysis was conducted using 1-way ANOVA and uncertainty is expressed as SEM (*P < 0.05, **P < 0.01, ***P < 0.001).

As TALK-1 in part controls β-cell [Ca^2+^]_i_ oscillation frequency and GSCI, we examined the role of the channel in cytokine-mediated changes to islet Ca^2+^ handling^14,26,27^. Oscillations in [Ca^2+^]_i_ were slower in WT than in TALK-1 KO islets (WT: 1.18 ± 0.07 oscillations/min and TALK-1 KO: 1.51 ± 0.16 oscillations/min, Fig. 2a–d, P < 0.01) and cytokine exposure accelerated [Ca^2+^]_i_ oscillations in both equivalently (WT: 2.01 ± 0.15x, P < 0.001 and TALK-1 KO: 1.77 ± 0.20x, P < 0.001). Cytokine exposure increased basal [Ca^2+^]_i_ in WT islets (11.36 ± 2.53% increase, Fig. 2e, P < 0.01) while basal [Ca^2+^]_i_ in TALK-1 KO islets was trending higher (6.15 ± 4.08% increase, Fig. 2e, P = 0.085). Cytokine exposure reduced 1^st^ phase GSCI in WT and TALK-1 KO islets (WT: 64.55 ± 5.99% decrease and TALK-1 KO: 68.12 ± 4.79% decrease, Fig. 2f, P < 0.001). In the absence of cytokines 2^nd^ phase GSCI was higher in TALK-1 KO islets than in WT islets (48.31 ± 19.55% increase, Fig. 2g, P < 0.05). Following cytokine exposure 2^nd^ phase GSCI in TALK-1 KO islets was reduced to WT levels (60.03 ± 4.89% decrease, Fig. 2g, P < 0.001) while 2^nd^ phase GSCI in WT islets was unaffected.

### Cytokine-mediated downregulation of TALK-1 increases β-cell [Ca^2+^]_ER_ storage

**Figure 3:**
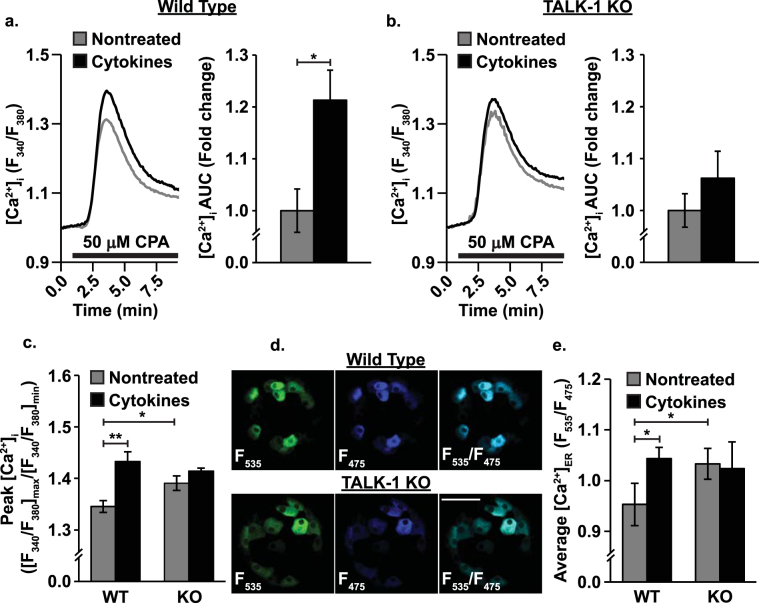
Cytokine exposure increases β-cell [Ca^2+^]_ER_. (**a**) Representative Fura-2 AM CPA responses of nontreated (gray) and cytokine treated (black) WT mouse β-cells (left; black bars correspond to the addition of CPA) and relative cytokine-induced change in [Ca^2+^]_i_ AUC (right; N = 3 animals), (**b**) representative Fura-2 AM CPA responses of nontreated (gray) and cytokine treated (black) TALK-1 KO mouse β-cells (left) and relative cytokine-induced change in [Ca^2+^]_i_ AUC (right; N = 3 animals), (**c**) average peak CPA responses ([F_340/380_]_max_/F[_340/380_]_min)_ for nontreated (gray) and cytokine treated (black) WT and TALK-1 KO mouse β-cells (N = 3 animals), (**d**) representative fluorescent images of the D4ER genetically encoded, β-cell specific [Ca^2+^]_ER_ indicator in WT (top) and TALK-1 KO (bottom) islets (green: F_535_, blue: F_475_, cyan: merge; scale bar is 50 µm), and (**e**) average D4ER intensity (F_535_/F_475_) of WT and TALK-1 KO mouse β-cells from intact islets (N = 4 animals). Statistical analysis was conducted using 1-way ANOVA and uncertainty is expressed as SEM (*P < 0.05, **P < 0.01).

Cytokines alter β-cell [Ca^2+^]_ER_ in addition to [Ca^2+^]_i_^5^. Because TALK-1 modulates [Ca^2+^]_ER_, we examined whether a cytokine-mediated effect on the channel tunes β-cell [Ca^2+^]_ER_^27^. First, [Ca^2+^]_ER_ was examined by blocking SERCA with CPA to induce [Ca^2+^]_ER_ release, then [Ca^2+^]_i_ was quantified. Under these conditions peak [Ca^2+^]_ER_ release was higher in TALK-1 KO than in WT β-cells (3.37 ± 1.37% increase, Fig. 3a–c, P < 0.05). Cytokine exposure increased peak [Ca^2+^]_ER_ release in WT (6.42 ± 2.12% increase, Fig. 3c, P < 0.05) but not TALK-1 KO β-cells. This was verified in whole islets using an adenoviral-based genetically-encoded, β-cell specific (under an insulin promoter) ER Ca^2+^ indicator (D4ER, Fig. 3d). This approach also indicated that [Ca^2+^]_ER_ in TALK-1 KO β-cells was higher in than in WT β-cells (9.12 ± 2.36% increase, Fig. 3e, P < 0.05). Furthermore, cytokine exposure again increased [Ca^2+^]_ER_ in WT (10.44 ± 3.57% increase, Fig. 3e, P < 0.05) but not TALK-1 KO β-cells.

### Cytokine exposure depolarizes β-cell *V*_m_ and increases electrical activity

**Figure 4:**
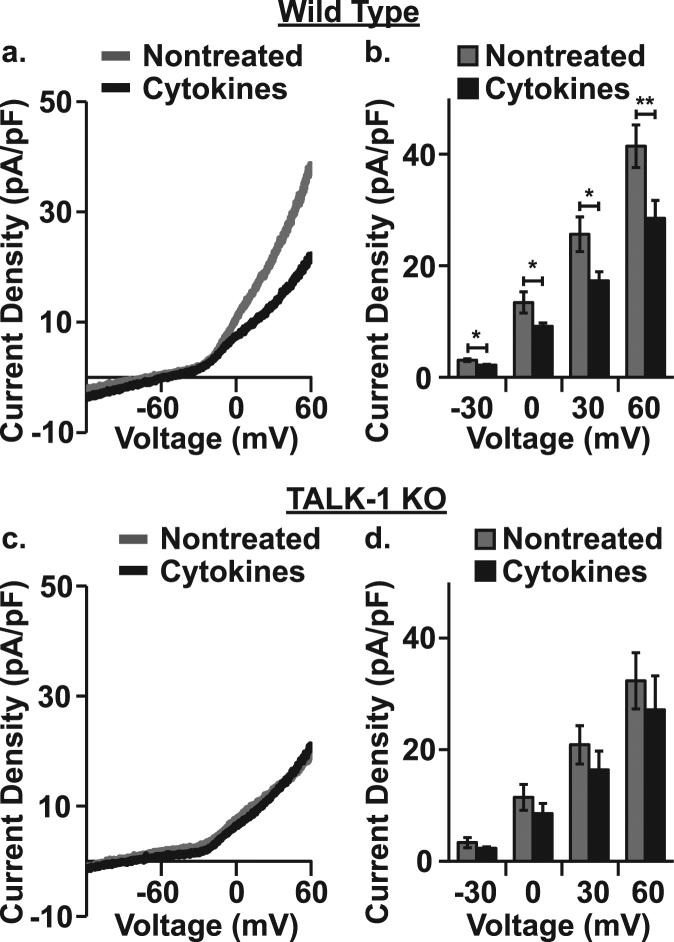
Cytokine exposure decreases TALK-1 K2P currents. (**a**) Representative whole-cell voltage-clamp recordings of K2P currents in nontreated (gray) and cytokine treated (black) WT mouse β-cells, (**b**) average K2P currents in nontreated (gray) and cytokine treated (black) WT mouse β-cells at selected voltages (N = 4 animals), (**c**) representative whole cell voltage ramp recordings of K2P currents in nontreated (gray) and cytokine treated (black) TALK-1 KO mouse β-cells, and (**d**) average K2P currents in nontreated (gray) and cytokine treated (black) TALK-1 KO mouse β-cells at selected voltages (N = 3 animals). Statistical analysis was conducted using paired two-tailed t-tests and uncertainty expressed as SEM (*P < 0.05, **P < 0.01).

To further probe cytokine-mediated TALK-1 channel effects on β-cell function, β-cell K2P currents and *V*_m_ were determined. For K2P current recording a whole-cell voltage-clamp technique was employed. β-cell *V*_m_ was ramped from −120 mV to 60 mV and the resulting whole-cell β-cell currents were measured. K2P currents were isolated by blocking K_ATP_ currents with tolbutamide, K_V_ currents with TEA, and K_Ca_ currents by removing Ca^2+^ and including EGTA in the bath solution. Cytokine exposure reduced K2P currents in WT (Δ_−30 mV_: −0.90 ± 0.30 pA/pF, Δ_0 mV_: −4.26 ± 1.97 pA/pF, Δ_30 mV_: −8.33 ± 3.50 pA/pF, Δ_60 mV_: −12.94 ± 5.00 pA/pF, Fig. 4a, b, P < 0.05) but not TALK-1 KO β-cells (Fig. 4c, d). The whole-cell K2P conductance of WT and TALK-1 KO β-cells was also analyzed between 0 and 60 mV. Cytokine exposure decreased WT β-cell K2P conductance (nontreated: 0.46 ± 0.02 pS/pF and cytokine treated: 0.31 ± 0.02 pS/pF, P < 0.001) but had no effect on TALK-1 KO β-cell K2P conductance (nontreated: 0.32 ± 0.01 pS/pF and cytokine treated: 0.31 ± 0.04 pS/pF). Together, these data indicate that cytokine exposure affects β-cell K2P currents in a TALK-1 specific manner.

**Figure 5:**
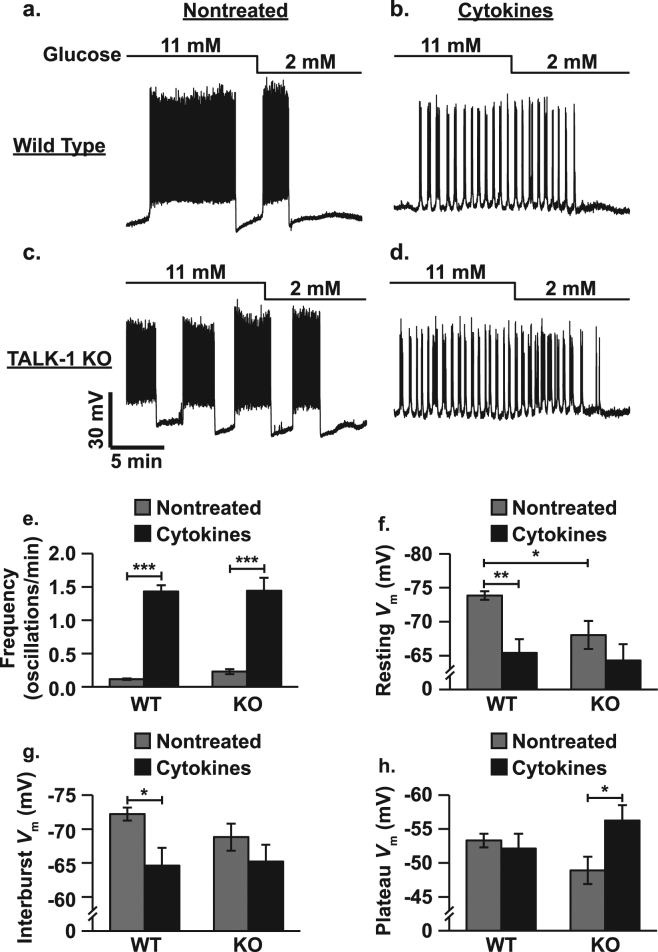
Cytokine exposure increases β-cell electrical excitability. (**a**) Representative perforated-patch current-clamp *V*_m_ recording of a nontreated WT β-cell (lines above figure indicate glucose concentrations), (**b**) representative *V*_m_ recording of a cytokine treated WT β-cell, (**c**) representative *V*_m_ recording of a nontreated TALK-1 KO β-cell, (**d**) representative *V*_m_ recording of a cytokine treated TALK-1 KO β-cell, (**e**) average electrical oscillation frequency for nontreated (gray) and cytokine treated (black) WT and TALK-1 KO β-cells (N ≥ 6 cells), (**f**) average resting *V*_m_ for nontreated (gray) and cytokine treated (black) WT and TALK-1 KO β-cells (N ≥ 8 cells), (**g**) average interburst *V*_m_ for nontreated (gray) and cytokine treated (black) WT and TALK-1 KO β-cells (N ≥ 7 cells), (**h**) average plateau *V*_m_ for nontreated (gray) and cytokine treated (black) WT and TALK-1 KO β-cells (N ≥ 8 cells). Statistical analysis was conducted using 1-way ANOVA and uncertainty is expressed as SEM (*P < 0.05, **P < 0.01, ***P < 0.001).

Changes in β-cell *V*_m_ were monitored in current-clamp mode using a perforated-patch technique to maintain the integrity of intracellular metabolism and signaling^30^. Cytokine exposure accelerated electrical oscillation frequency in WT β-cells more than in TALK-1 KO β-cells (WT: 12.29 ± 0.79-fold increase and TALK-1 KO: 6.27 ± 0.84-fold increase, Fig. 5a–e, P < 0.001). As the oscillation frequency of WT and TALK-1 KO islets were indistinguishable following cytokine exposure this difference was likely due to the fact that nontreated TALK-1 KO islets oscillate more rapidly than WT islets^14^. The impact of cytokine exposure on β-cell *V*_m_ was studied under basal (2 mM glucose, resting *V*_m_) and stimulatory conditions (11 mM glucose, interburst *V*_m_ (between plateau potentials) and plateau *V*_m_ (during plateau potentials)) in whole islets. Cytokines depolarized WT β-cell resting *V*_m_ (8.44 ± 2.12 mV, Fig. 5f, P < 0.01) and interburst *V*_m_ (7.60 ± 2.80 mV, Fig. 5g, P < 0.05) but not plateau *V*_m_. Cytokine exposure did not alter TALK-1 KO β-cell resting and interburst *V*_m_, but hyperpolarized plateau *V*_m_ (7.35 ± 3.03 mV, Fig. 5h, P < 0.05). TALK-1 KO β-cell resting *V*_m,_ was likely not affected by cytokine exposure because they are already more depolarized that WT β-cells (5.82 ± 2.16 mV, Fig. 5f, P < 0.05).

### Cytokine exposure reduces β-cell K_slow_ currents

**Figure 6:**
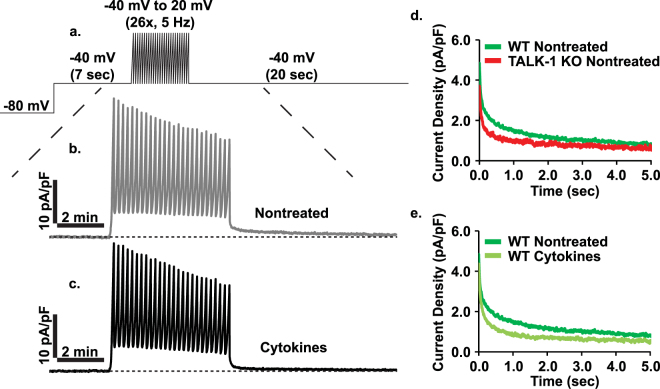
Cytokine exposure reduces β-cell K_slow_ currents. (**a**) Overview of K_slow_ perforated-patch voltage-clamp recording protocol, (**b**) representative nontreated WT β-cell K_slow_ trace (average of 6 cells from one animal), (**c**) representative cytokine treated WT K_slow_ trace (average of 6 cells from one animal), (**d**) average K_slow_ currents for nontreated WT (dark green) and TALK-1 KO (red) β-cells (N ≥ 15 cells), and (**e**) average K_slow_ currents for nontreated (dark green) and cytokine treated (light green) WT β-cells (N ≥ 31 cells).

As K_slow_ also contributes to islet [Ca^2+^]_i_ oscillations and is controlled by [Ca^2+^]_i_ influx as well as [Ca^2+^]_ER_ release, this current was next investigated as a possible contributor to cytokine-mediated elevated β-cell electrical excitability^16,19–22^. As previously described, Ca^2+^ influx was activated using a voltage command to sequentially generate 26 APs, then the subsequent outward K^+^ currents were recorded (Fig. 6a–c)^19^. K_slow_ currents were fit to a model of two-phase exponential decay using GraphPad Prism (Table 1) and the related kinetic parameters were determined (Y_0_: peak K_slow_ amplitude, % fast: % of K_slow_ occurring in the fast phase, K_f_: fast phase rate constant, K_s_: slow phase rate constant, t_1/2, f_: fast phase half-life, t_1/2, s_: slow phase half-life, τ_f_: fast phase time constant, and τ_s_: slow phase time constant). The resulting K_slow_ currents were biphasic, composed of a rapidly decaying fast phase followed by a gradually declining slow phase (Fig. 6d, e). In the absence of cytokines peak K_slow_ amplitude was lower in TALK-1 KO β-cells and the current decayed more rapidly than in WT β-cells. In WT β-cells cytokine exposure reduced peak K_slow_ amplitude and accelerated decay of the current. This reduction in K_slow_ would be predicted to increase β-cell electrical excitability^22^.

**Table 1:** Kinetic parameters for the two-phase exponential decay of K_slow_.

Parameter	(A) WT Nontreated (N = 43)	(B) WT Cytokines (N = 31)	(C) TALK-1 KO Nontreated (N = 15)	P-value A vs B	P-value A vs C	P-value B vs C
Y_0_	4.427 ± 0.028	4.100 ± 0.026	3.418 ± 0.030	***	***	***
% fast	58.80 ± 0.39	68.58 ± 0.48	72.71 ± 0.43	***	***	***
K_f_	14.26 ± 0.35	14.20 ± 0.32	13.94 ± 0.39	ns	ns	ns
K_s_	0.778 ± 0.012	1.301 ± 0.026	0.669 ± 0.024	***	**	***
t_1/2, f_	0.049 ± 0.001	0.049 ± 0.001	0.050 ± 0.001	ns	ns	ns
t_1/2, s_	0.892 ± 0.014	0.533 ± 0.011	1.037 ± 0.038	***	***	***
τ_f_	0.070 ± 0.002	0.070 ± 0.002	0.072 ± 0.002	ns	ns	ns
τ_s_	1.286 ± 0.020	0.769 ± 0.015	1.496 ± 0.055	***	***	***

Statistical analysis was conducted using 1-way ANOVA and uncertainty is expressed as SEM (**P < 0.01, ***P < 0.001).

### Cytokine-induced loss of TALK-1 diminishes insulin expression and eventually results in reduced GSIS

**Figure 7:**
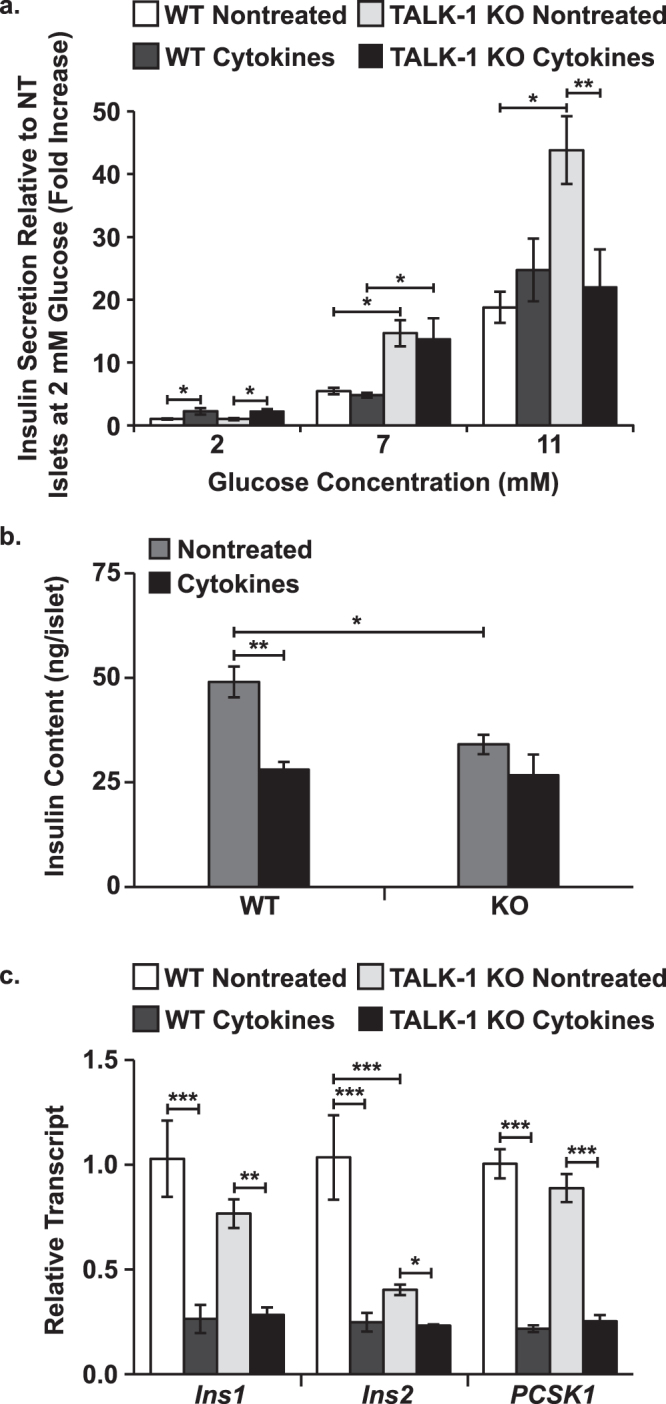
Cytokine exposure decreases insulin protein and related transcripts in WT and TALK-1 KO islets but only disrupts GSIS from TALK-1 KO islets. (**a**) Average basal (2 mM glucose) and glucose-stimulated (7 and 11 mM glucose) insulin secretion from nontreated and cytokine treated WT and TALK-1 KO islets (N ≥ 3 animals), (**b**) average insulin content of nontreated (gray) and cytokine treated (black) WT and TALK-1 KO islets (N = 3 animals), and (**c**) preproinsulin (*Ins1* and *Ins2*) and proprotein convertase 1 (*PCSK1*) transcript relative to *GAPDH* in nontreated and cytokine treated WT and TALK-1 KO islets (N = 3 animals). Statistical analysis was conducted using 1-way ANOVA and uncertainty is expressed as SEM (*P < 0.05, **P < 0.01, ***P < 0.001).

Cytokine-mediated effects on GSIS were investigated. Data are presented as a fold increase compared to insulin secretion from nontreated islets at 2 mM glucose (un-normalized data shown for nontreated islets at 2 mM glucose). Insulin secretion at 2 mM glucose was indistinguishable in WT and TALK-1 KO islets (WT 7.26 ± 1.19 pg/islet/hr and TALK-1 KO: 7.67 ± 0.84 pg/islet/hr) and cytokine exposure increased basal secretion equivalently in WT and TALK-1 KO islets (WT: 2.24 ± 0.52x, P < 0.05 and TALK-1 KO: 2.23 ± 0.35x, Fig. 7a, P < 0.05). Under stimulatory glucose conditions (7 and 11 mM glucose) TALK-1 KO islets secreted more insulin than WT islets (7 mM glucose- WT: 5.47 ± 0.52x and TALK-1 KO: 14.67 ± 2.11x, P < 0.05; 11 mM glucose- WT: 18.78 ± 2.49x and TALK-1 KO: 43.83 ± 5.40x, Fig. 7a, P < 0.05). Cytokine exposure had no effect on GSIS from WT or TALK-1 KO islets at 7 mM glucose but reduced GSIS from TALK-1 KO islets at 11 mM glucose (22.02 ± 6.00x, Fig. 7a, P < 0.01). Next, insulin protein and transcript levels were investigated to determine whether the cytokine-induced reduction in GSIS from TALK-1 KO islets was due to reduced insulin expression or defective secretion. TALK-1 KO islets contained less insulin protein than WT islets (WT: 49.03 ± 3.68 ng insulin/islet and TALK-1 KO: 34.08 ± 2.31 ng insulin/islet, Fig. 7b, P < 0.05). *Ins2* transcript was also lower in TALK-1 KO islets (61.06 ± 6.19% decrease, Fig. 7c, P < 0.01) while *Ins1* and proprotein convertase 1 (*PCSK1*) transcript levels were indistinguishable in WT and TALK-1 KO islets. The insulin content of WT islets decreased to a level similar to TALK-1 KO islets following cytokine exposure (28.09 ± 1.80 ng insulin/islet, Fig. 7b, P < 0.01) but did not change in TALK-1 KO islets. After cytokine exposure *Ins1*, *Ins2*, and *PCSK1* transcripts decreased to similar levels in both WT and TALK-1 KO islets (Fig. 7c, P < 0.05). These results suggest that decreased GSIS from TALK-1 KO islets is due to a dysregulation of insulin secretion rather than insulin expression.

### Statistical Analysis

All experimental data presented here are shown as mean values ± SEM. Unpaired two-tailed t-tests, paired two-tailed t-tests, and 1-way analysis of variance (ANOVA) tests were used to determine statistical significance between test groups as appropriate. P < 0.05 was considered statistically significant and P < 0.10 was considered to be trending toward significance.

## Discussion

Here we demonstrated that both K_slow_ and TALK-1 currents are important contributors to cytokine-mediated changes in β-cell electrical activity and Ca^2+^ handling. Cytokine exposure reduced K_slow_, which was accompanied by increased electrical excitability and [Ca^2+^]_i_ oscillation frequency. These cytokine-induced changes were caused in part by reduced [Ca^2+^]_ER_ release, which resulted from diminished TALK-1 expression. Moreover, during low-grade inflammation, downregulation of TALK-1 preserved GSIS presumably by maintaining 2^nd^ phase GSCI. However, β-cells without functional TALK-1 channels failed to undergo cytokine-induced compensatory Ca^2+^ changes leading to defective GSCI and GSIS. Together these data suggest that TALK-1 and K_slow_ play important roles in β-cell adaptation to acute low-grade inflammation.

Although it has been established that low-grade inflammation perturbs β-cell Ca^2+^ handling, the electrical signaling mechanisms responsible for these changes have not been characterized^5–7,13^. Many ion channels contribute to physiological control of β-cell electrical excitability and some of these channels become perturbed during conditions of low-grade inflammation^5,6,28,31^. For example, TALK-1 K^+^ flux plays a key role in modulating β-cell electrical excitability and expression of the gene that encodes TALK-1 (*KCNK16*) is reduced during inflammation^14,28^. As predicted based on knockout of the channel, cytokine-mediated loss of TALK-1 currents depolarized β-cell *V*_m_, which was accompanied by elevated electrical and [Ca^2+^]_i_ oscillation frequency. TALK-1 KO β-cells displayed a similar cytokine-induced increase in excitability, but *V*_m_ was not changed and 2^nd^ phase GSCI was decreased. The frequency of β-cell [Ca^2+^]_i_ oscillations controls insulin secretion^32^. Therefore, by lowering TALK-1 expression β-cells were able to increase [Ca^2+^]_i_ oscillation frequency and acutely maintain physiological [Ca^2+^]_i_ levels during 2^nd^ phase GSCI in response to low-grade inflammation^33,34^. However, when [Ca^2+^]_i_ oscillations become too rapid (e.g. in TALK-1 KO islets following cytokine exposure) the amplitude of [Ca^2+^]_i_ oscillations begins to decrease^35^. Thus, in TALK-1 KO islets [Ca^2+^]_i_ levels declined during 2^nd^ phase GSCI along with [Ca^2+^]_i_ oscillation amplitude. Disruption of islet [Ca^2+^]_i_ oscillations is a hallmark of metabolic stress in obese rodent models like those on a high fat diet (HFD) or without leptin receptors^36,37^. The deterioration of orderly [Ca^2+^]_i_ oscillations in islets is also believed to contribute to the loss of pulsatile insulin secretion observed in T2DM, which leads to insulin resistance and hyperglycemia^38,39^. Thus, β-cells accelerated [Ca^2+^]_i_ oscillations to maintain GSCI during acute low-grade inflammation; however, with chronic inflammation the Ca^2+^ response continued to change, eventually resulting in reduced islet [Ca^2+^]_i_ and GSIS. Although TALK-1 plays a role in the adaptation of β-cells to low-grade inflammation, [Ca^2+^]_i_ oscillations were still accelerated in TALK-1 KO islets following cytokine exposure, which implicates other ion channels in these cytokine-induced β-cell effects.

Activation of a K_slow_ current is the primary contributor to β-cell *V*_m_ repolarization following the slow waves of depolarization that mediate [Ca^2+^]_i_ oscillations^16,19–22^. Following cytokine exposure β-cell *V*_m_ became depolarized during the electrically silent periods between plateau potentials and the frequency of electrical oscillations increased. This suggests that cytokines affect one or more K_Ca_ channels that make up the K_slow_ current. Indeed, pharmacological inhibition of K_slow_ accelerates islet [Ca^2+^]_i_ oscillations similar to cytokine exposure^22^. As anticipated, our results showed that low level cytokine exposure was sufficient to significantly reduce the magnitude and duration of K_slow_ in WT β-cells. The K_slow_ current was biphasic in nature, likely because the current is comprised of multiple K^+^ channels with varying degrees of Ca^2+^ sensitivity as well as differing activation and inactivation kinetics. Furthermore, it has been established that [Ca^2+^]_ER_ release contributes significantly to K_slow_ activation^21^. Therefore, K_slow_ kinetics may be influenced by the temporal profile of ER Ca^2+^-induced Ca^2+^ release (CICR) or the proximity of K_Ca_ channels to [Ca^2+^]_ER_ release sites^40^. TALK-1 has been shown to act as a counter-current for Ca^2+^ release from the ER of β-cells and indeed genetic ablation of TALK-1 increases β-cell [Ca^2+^]_ER_ storage and reduces the magnitude of K_slow_^27^. Thus, cytokine-mediated reduction in ER TALK-1 likely contributes to reduced K_slow_ currents by preventing efficient release of Ca^2+^ from ER stores. Likewise, under conditions of β-cell stress (e.g. low-grade inflammation) SERCA expression is reduced, which would be expected to perturb [Ca^2+^]_ER_ and further diminish K_slow_ currents^19–21,41^. Cytokine-induced reduction of K_slow_ caused more frequent and shorter interburst silent phases in β-cell electrical activity during glucose stimulation. Due to these effects on electrical activity, islet [Ca^2+^]_i_ oscillations were accelerated, amplitude was reduced, and [Ca^2+^]_i_ was elevated at the nadir between oscillations. In the future, it will be important to identify the K_Ca_ channel(s) responsible for the reduction in K_slow_ currents following cytokine exposure and to determine if these channels can be manipulated to improve β-cell function during low-grade inflammation present during the pathogenesis of T2DM.

Because [Ca^2+^]_ER_ homeostasis is essential for many biological processes including protein expression, folding, and transport, dysregulation of this equilibrium could also lead to unfolded protein response (UPR) and ER stress^42^. In fact, pro-inflammatory cytokine exposure has been shown to cause ER stress, hence it is plausible that cytokine-induced disruption of [Ca^2+^]_ER_ handling contributes to β-cell dysfunction and destruction^43^. Interestingly, the islets of TALK-1 KO mice on a long-term (20 week) HFD displayed reduced ER stress markers^27^. Cytokine exposure increased [Ca^2+^]_ER_ levels to those observed in TALK-1 deficient β-cells, which may help mitigate β-cell ER stress during acute low-grade inflammation. However, it is well-established that prolonged cytokine exposure depletes β-cell [Ca^2+^]_ER_ levels^6,7,44^. This results in part from diminished islet SERCA expression observed under low-grade inflammatory conditions and in T2DM patients^45,46^. Reduced SERCA expression should reduce [Ca^2+^]_ER_ refilling and thus lower [Ca^2+^]_ER_ stores. These data suggest that loss of TALK-1 expression preserves [Ca^2+^]_ER_ levels during acute inflammation, but may not compensate for the loss of [Ca^2+^]_ER_ uptake during chronic low-grade inflammation. Further studies are required to fully determine the importance of TALK-1-mediated elevation of β-cell [Ca^2+^]_ER_ levels in response to acute and chronic low-grade inflammation.

In addition to accelerating islet [Ca^2+^]_i_ and electrical oscillations, low-grade inflammation resulted in increased basal [Ca^2+^]_i_ at 2 mM glucose and decreased 1^st^ phase GSCI at 11 mM glucose. While these changes in β-cell Ca^2+^ handling could be partly due to impaired [Ca^2+^]_ER_ uptake (e.g. reduced SERCA activity), other mechanisms are anticipated to play a significant role^5–7^. For example, cytokine exposure diminishes mitochondrial ATP production, which could decrease 1^st^ phase GSCI by enhancing K_ATP_ channel activity and thus limiting β-cell *V*_m_ depolarization^29,47^. Cytokine exposure also activates voltage-dependent Ca^2+^ channels (VDCCs), which would be predicted to elevate basal β-cell [Ca^2+^]_i_ levels^5,6,31^. This suggests that the global changes in ion channel activity (e.g. K_ATP_ and VDCC activation) only modestly depolarize β-cell *V*_m_. However, cytokine-induced depolarization of β-cells at rest (2 mM glucose) diminishes glucose-stimulated *V*_m_ depolarization. This decreased glucose-stimulated Δ*V*_m_ leads to reduced GSCI following cytokine exposure. Interestingly, islets of T2DM patients display elevated basal insulin secretion and a defective 1^st^ phase GSIS response, which would be projected to result from increased basal islet [Ca^2+^]_i_ and decreased 1^st^ phase GSCI^48,49^. In the future, it will be important to determine how these cytokine-mediated changes to islet Ca^2+^ handling contribute to perturbations in GSIS during the pathogenesis of T2DM.

Not only do cytokines affect β-cell electrical excitability and Ca^2+^ handling they also modulate insulin expression and secretion^50,51^. Genetic ablation of islet TALK-1 augments GSIS by depolarizing β-cell *V*_m_, thus it was theorized that a cytokine-mediated drop in TALK-1 expression would also increase GSIS^14^. Acute cytokine exposure increased basal insulin secretion at 2 mM glucose, which is likely due to elevated basal [Ca^2+^]_i_ resulting in part from VDCC activation^5,6,31^. GSIS from WT islets was unaffected by acute cytokine exposure, but GSIS from TALK-1 KO islets was decreased by approximately 50% at 11 mM glucose. This suggests that acute augmentation of β-cell [Ca^2+^]_i_ by reducing TALK-1 expression maintains physiological GSIS following cytokine exposure. However, this adaptive GSIS response becomes less robust when TALK-1 is absent for an extended period of time (i.e. TALK-1 KO), which impairs 2^nd^ phase GSCI and GSIS. TALK-1 KO β-cells may compensate for channel loss over time, and as a result they become more sensitive to low-grade inflammation. TALK-1 KO β-cells are also more sensitive to glucose, displaying higher GSIS at lower glucose concentrations^14^. Thus, it is possible that the readily releasable insulin granule pool was depleted under these conditions, resulting in decreased GSIS. Interestingly, without cytokines TALK-1 KO islets contained less insulin protein and *Ins2* transcript than WT islets even though their GSIS was greater. The enhanced secretory ability of TALK-1 KO islets likely feeds back and limits insulin expression. As insulin content becomes equivalent in WT and TALK-1 KO islets following cytokine exposure, decreased GSIS from TALK-1 KO islets was not likely due to defective insulin expression. While WT islets responded to cytokines with increased secretory capacity, TALK-1 KO islets may have been maximally active and thus not capable of further amplifying GSIS. It is probable that changes in islet GSIS under acute low-grade inflammatory conditions are related to the Ca^2+^-modulatory function of islet TALK-1 and K_slow_ currents. By downregulating TALK-1 channels and inhibiting K_Ca_ channel activity, normal GSIS is maintained in the short-term.

In conclusion, K_slow_ currents are reduced during low-grade inflammation resulting in increased β-cell electrical and [Ca^2+^]_i_ oscillation frequency. Cytokine-induced downregulation of TALK-1 contributes to decreased K_slow_ activity by reducing [Ca^2+^]_ER_ release. Importantly, these changes acutely preserve normal β-cell [Ca^2+^]_i_ levels during 2^nd^ phase GSCI and thus help maintain GSIS during low-grade inflammation. These results also predict that during chronic inflammation loss of TALK-1 impairs β-cell Ca^2+^ handling resulting in defective GSIS.

## Methods

### Materials and reagents

Unless otherwise stated all research materials were obtained from Thermo Fisher Scientific and Sigma-Aldrich.

### Mouse islet and β-cell isolation and culture

Animals were handled in compliance with the *Guide for the care and use of laboratory animals*, Eighth edition (2011) (http://grants.nih.gov/grants/olaw/guide-for-the-care-and-use-of-laboratory-animals.pdf) and in line with guidelines approved by the Vanderbilt University Animal Care and Use Committee (protocol # M1600063-00). Wild type (WT) and TALK-1 knockout (KO) islets were isolated from the pancreata of age-matched 6- to 12-week old C57B16/J mice as previously described^52^. Islets were dispersed using 0.0075% trypsin-EDTA and cultured overnight on poly-d-lysine coated 35 mm glass-bottom dishes in RPMI 1640 supplemented with 15% FBS, 100 IU∙ml^−1^ penicillin, and 100 mg∙ml^−1^ streptomycin (islet media) at 37 °C, 5% CO_2_. Alternatively, whole islets were cultured overnight on poly-d-lysine coated glass-bottom dishes in islet media. Unless otherwise specified, after overnight incubation all samples were supplemented with either fresh islet media or islet media with cytokines (0.1 ng/mL TNF-α, 0.05 ng/mL IL-1β, and 10 ng/mL IFN-γ) and used for testing within 48 hrs.

### Mouse islet qRT-PCR

WT or TALK-1 KO mouse islets were cultured for 24 hrs at 37 °C, 5% CO_2_ in 6-cm petri dishes in islet media or in islet media with cytokines. RNA was isolated with TRIzol Reagent as per manufacturer’s instructions. Islet cDNA was prepared from RNA with SuperScript IV Reverse Transcriptase using an S1000 Thermal Cycler (Bio-Rad). Transcript abundance relative to GAPDH was determined by qRT-PCR using primers specific to TALK-1 (*KCNK16*; forward, 5′-CAG CTC TGG CTG CTC AGT AGG-3′; reverse, 5′-CTC ATG CAG AGA TGG GGA TCT T-3′), SERCA2b (*ATP2A2*; forward, 5′-AGG GAC TGC AGT GGC TAA GA-3′; reverse, 5′-GCC ACA ATG GTG GAG AAG TT-3′), preproinsulin 1 (*Ins1*; forward, 5′-AGC AAG CAG GTC ATT GTT CC-3′; reverse, 5′-GAC GGG ACT TGG GTG TGT AG-3′), preproinsulin 2 (*Ins2*; forward, 5′-TCT TCT ACA CAC CCA TGT CCC-3′; reverse, 5′-GGT GCA GCA CTG ATC CAC-3′), proprotein convertase 1 (*PCSK1*; forward, 5′-CCT CCT ACA GCA GTG GTG ATT ACA-3′; reverse, 5′-GGG TCT CTG TGC AGT CAT TGT-3′), and GAPDH (forward, 5′-GAG AAT GGG AAG CTT GTC ATC AAC-3′; reverse, 5′-ACT CCA CGA CAT ACT CAG CAC CAG-3′ using iTaq Universal SYBR Green Supermix (Bio-Rad) with a CFX Real time PCR Instrument (Bio-Rad).

### Human islet western blotting

Human islets were provided through an approved protocol by the Integrated Islet Distribution Program (IIDP) (https://iidp.coh.org/). The IIDP obtained informed consent for deceased donors in accordance with NIH guidelines prior to reception of human islets for our studies. Work detailed here was approved by the Vanderbilt University Health Sciences Committee Institutional Review Board (IRB# 110164). Islet donor information is provided in Table 2. The islets were cultured overnight in islet media or islet media with cytokines. The islets were washed with ice cold phosphate buffered saline (PBS) and resuspended in ice cold radioimmunoprecipitation buffer (RIPA) with (in mmol/L) 50.0 Tris pH 7.4, 150.0 NaCl, and 1.0 EDTA (Corning) with 1.0 (w/v%) NP-40 and 1x protease inhibitor cocktail (Roche, Penzberg, Germany). Islets were sonicated on ice with a sonic dismembrator model 100, incubated at 4 °C for 30 min, and centrifuged for 10 min at 12,000 RPM, 4 °C. Protein concentration was determined by BCA assay as per manufacturer’s instructions. Samples (40 µg/lane) were resolved under denaturing conditions on a NuPAGE 4–12% Bis-Tris Gel then transferred to a nitrocellulose membrane. The membrane was incubated with rabbit anti-TALK-1 (1:500) overnight at 4 °C then TALK-1 bands were visualized with HRP-conjugated donkey anti-rabbit secondary (1:1500; Jackson ImmunoResearch Laboratories) and SuperSignal™ West Pico Chemiluminescent Substrate with a Bio-Rad ChemiDoc Imaging System.

**Table 2:** Human islet donor information. Summary of relevant information for human donors of islets used for immunofluorescence microscopy and immunoblotting.

Donor	1	2	3
Age	24	41	27
BMI	34.8	28.5	31.2
Sex	Male	Male	Female
Ethnicity	Caucasian	Caucasian	Caucasian
HbA1c	5.4%	5.3%	5.6%

### Mouse and human islet immunofluorescent analysis

WT mouse islets or human islets were cultured overnight in islet media or islet media with cytokines. The islets were washed with cold PBS and resuspended in 150 µL of 2.25 mg/mL rat tail collagen I supplemented with 1x DMEM, 200 mM HEPES, and 75 mg/mL sodium bicarbonate on ice. The samples were incubated for 40 min in a 96 well-plate at 37 °C, 5% CO_2_ to cross-link the collagen mixture. The collagen-embedded islets were fixed for 10 min on ice with 4% paraformaldehyde (PFA, Electron Microscopy Sciences), then the PFA was removed and the gels were transferred to 3 mL of cold PFA in a 12 well plate for 15 min (Corning). After fixation the gels were embedded in paraffin and processed into 5 µm slices. Rehydrated slices were stained with rabbit anti-TALK-1 (1:175) and guinea pig anti-insulin (1:500, Dako) followed by secondary antibodies (1:500 anti-rabbit Alexa Fluor 488 and 1:500 anti-guinea pig Alexa Fluor 647 (Jackson ImmunoResearch Laboratories)). Immunofluorescence was imaged using a Nikon Eclipse TE2000-U microscope equipped with an epifluorescence illuminator (Sutter Instrument Company), a CCD camera (HQ2; Photometrics, Inc), and Nikon Elements software (Nikon, Inc). TALK-1 immunofluorescence was quantified using the ImageJ Fiji image processing pack.

### Fura-2 AM imaging of islet glucose-stimulated Ca^2+^ influx

WT and TALK-1 KO mouse islets were loaded with 2 µM Fura-2-acetoxymethyl ester (AM) for 25 min at 37 °C, 5% CO_2_. The islets were washed twice with Krebs-Ringer–HEPES buffer (KRHB) with (in mmol/L) 119.0 NaCl, 2.0 CaCl_2_, 4.7 KCl, 25.0 HEPES, 1.2 MgSO_4_, 1.2 KH_2_PO_4_ (adjusted to pH 7.4 with NaOH) then incubated 20 min in KRHB with 2.0 mM glucose (KRHB-2mM) at 37 °C, 5% CO_2_. Islet [Ca^2+^]_i_ was measured every 5 sec as a ratio of Fura-2 AM fluorescence at 340 and 380 nm (F_340_/F_380_) with a Nikon Eclipse TE2000-U microscope and the data was analyzed using Nikon Elements software. The islets were perifused at 37 °C with a flow of 2 mL/min KRHB-2mM for 2 min then perifused under identical conditions with KRHB with 11.0 mM glucose (KRHB-11mM) for 30 min.

### Fura-2 AM imaging of β-cell [Ca^2+^]_ER_

WT and TALK-1 KO mouse islet cells were loaded with Fura-2 AM, washed twice with Ca^2+^-free KRHB-11mM with 125 µM diazoxide (Enzo), then incubated 10 min in Ca^2+^-free KRHB-11mM with 125 µM diazoxide. Islet [Ca^2+^]_i_ was measured every 5 sec as a ratio of Fura-2 AM fluorescence at 340 and 380 nm (F_340_/F_380_) with a Nikon Eclipse TE2000-U microscope and the data was analyzed using Nikon Elements software. The cells were perifused at 37 °C with a flow of 2 mL/min KRHB-11mM with 100 µM diazoxide for 2 min then perifused with KRHB-11mM with 100 µM diazoxide and 50 µM cyclopiazonic acid (CPA) for 3 min to release [Ca^2+^]_ER_ into the cytoplasm. Flow was then stopped and the Fura-2 AM imaging allowed to continue for 15 min.

### D4ER imaging of β-cell [Ca^2+^]_ER_

WT and TALK-1 KO mouse islets were cultured for 2 hrs with a 1000x multiplicity of inflection (MOI) of the ER-targeted Ca^2+^ probe D4ER then transferred to islet media in poly-D-lysine coated glass-bottom dishes for 48 hrs at 37 °C, 5% CO_2_. After 48 hrs, the islet media was replaced with fresh islet media or islet media with cytokines and the islets were cultured an additional 24 hrs at 37 °C, 5% CO_2_. The islets were washed twice with KRHB-11mM with 100 µM diazoxide then incubated 10 min in KRHB-11mM with 100 µM diazoxide. [Ca^2+^]_ER_ concentrations were measured every minute as a ratio of D4ER fluorescence at 535 and 475 nm (F_535_/F_475_) using a Zeiss Confocal Laser Scanning Microscope equipped with Zeiss ZEN software.

### β-cell voltage clamp recordings

WT and TALK-1 KO mouse islet cells were washed twice with KRHB-11mM. A whole-cell patch clamp technique was employed to record TALK-1-like K2P currents in single β-cells using an Axopatch 200B amplifier in voltage clamp mode with pCLAMP10 software (Molecular Devices). Cells were patched in KRBS-11mM, then in order to isolate K2P channel currents flow was switched to Ca^2+^-free KRHB-11mM with (in mmol/L) 0.2 tolbutamide (MP Biomedicals), 10.0 tetraethylammonium chloride hydrate (TEA), and 1.0 ethylene glycol-bis(β-aminoethyl ether)-N,N,N’,N’-tetraacetic acid (EGTA) (adjusted to pH 7.4 with NaOH). Patch electrodes (6–12 MΩ) were filled with voltage clamp intracellular solution (IC) with (in mmol/L) 140.0 KCl, 1.0 MgCl_2_, 10.0 EGTA, 10.0 HEPES, and 8.0 Mg-ATP (adjusted to pH 7.2 with KOH). For all voltage-clamp recordings the command voltage was held at −80 mV for 15 sec then increased from −120 mV to +60 mV over a period of 1 sec.

### β-cell *V*_m_ recordings

WT and TALK-1 KO mouse islet clusters were washed twice with KRHB-11mM then incubated in KRHB-11mM for 20 min. Patch electrodes (4–6 MΩ) were filled with *V*_m_ IC with (in mmol/L) 140.0 KCl, 1.0 MgCl_2_, and 5.0 HEPES (adjusted to pH 7.2 with KOH) supplemented with 20 µg/mL amphotericin B. The *V*_m_ of individual β-cells within islet clusters (10–20 cells) was recorded in current clamp mode using an Axopatch 200B amplifier with pCLAMP10 software. The electrical activity of patched β-cells was recorded for at least 10 min in KRHB-11mM then flow was changed to KRHB-2mM until action potential (AP) firing ceased for at least 3 min and baseline *V*_m_ stabilized. Cells were identified as β-cells if electrical activity ceased with 2 mM glucose.

### β-cell K_slow_ current recordings

Mouse islet cells were washed twice with KRHB-11mM. Single cells were patched in KRBS-11mM and a perforated patch clamp technique was employed to record β-cell K_slow_ currents using an Axopatch 200B amplifier with pCLAMP10 software. Patch electrodes (6–12 MΩ) were filled with K_slow_ IC with (in mmol/L) 76.0 K_2_SO_4_, 10.0 KCl, 10.0 NaCl, 1.0 MgCl_2_, and 5.0 HEPES (adjusted to pH 7.35 with KOH). For all K_slow_ electrophysiological recordings the command voltage was held at −40 mV for 7 sec, ramped between −40 and 20 mV for 5.2 sec (wave form: triangle, train rate: 5 Hz, pulse width: 0.2 sec), and returned to −40 mV for 20 sec.

### Islet insulin content and glucose-stimulated insulin secretion

WT and TALK-1 KO mouse islets were cultured overnight in islet media (supplemented with 0.5 mg/mL BSA) or islet media with cytokines at 37 °C, 5% CO_2_. Islets were cultured in equilibration media (DMEM (no glucose) with 10% FBS, 0.5 mg/mL BSA, 10 mM HEPES, and 0.5 mM CaCl_2_) supplemented with 5.6 mM glucose for 1 hr at 37 °C, 5% CO_2_. For insulin secretion, 20 islets were picked on ice into a 24-well plate (Corning) containing 500 µL of secretion media (DMEM (no glucose) with 0.5 mg/mL BSA, 10 mM HEPES, and 0.5 mM CaCl_2_) supplemented with 2 mM or 11 mM glucose, then insulin secretion was initiated for 1 hr at 37 °C, 5% CO_2_. After 1 hr, the plate was transferred to ice for 10 min to halt secretion and supernatants were collected in low retention 1.6 mL centrifuge tubes, which were stored at −20 °C until analyzed. Supernatants were supplemented with 1:100 mammalian protease inhibitor cocktail (MPIC). Whole islet insulin was extracted overnight at 4 °C into 400 µL of acid ethanol. Islet secretion supernatants and protein extracts were analyzed by the Vanderbilt Hormone Assay and Analytical Services Core (supported by NIH grants DK059637 and DK020593).

### Data Availability

All data are available upon request from the corresponding author.

## Electronic supplementary material

Supplemental Information
