# Supplementary material for: Cytokine-mediated changes in K+ channel activity promotes an adaptive Ca2+ response that sustains β-cell insulin secretion during inflammation
Source: Sci Rep. 2018 Jan 18;8:1158. doi: 10.1038/s41598-018-19600-x (PMC5773563; doi:10.1038/s41598-018-19600-x)
Supplement: Supplementary file 1 — Supplemental Information [file 41598_2018_19600_MOESM1_ESM.pdf]

SUPPLEMENTARY INFORMATION

Cytokine-mediated changes in K^+ channel activity promotes an adaptive Ca^{2+} response that sustains β-cell insulin secretion during inflammation

Matthew T. Dickerson, Avery M. Bogart, Molly K. Altman, Sarah C. Milian, Kelli L. Jordan, Prasanna K. Dadi, and David A. Jacobson

Table of Contents

1. Average of all whole-cell K2P current recordings for WT and TALK-1 KO β-cells……2
2. Annexin-V-FITC apoptosis assay for WT and TALK-1 KO β-cells……………..……….3
3. Quantification of $[Ca^{2+}]_i$ oscillation amplitudes in WT and TALK-1 KO β-cells…..…….5
4. Unprocessed human islet TALK-1 western blots……………………………………....6

1. Average of all whole-cell K2P current recordings for WT and TALK-1 KO β-cells

Average K2P current densities were calculated for nontreated WT (N=39 cells) and TALK-1 KO (N=31 cells) β-cells by averaging all whole-cell K2P voltage-clamp recordings for voltages between -120 mV and 60 mV (Fig. S1a). WT and TALK-1 KO K2P currents were compared at selected voltages (-30 mV, 0 mV, 30 mV, and 60 mV; Fig. S1b) using an unpaired two-tailed t-test. TALK-1 KO K2P current density was lower than WT K2P current density at 0 mV (WT: 13.01±0.48 pA/pF and TALK-1 KO: 10.73±0.49 pA/pF, P<0.01), 30 mV (WT: 25.68±0.99 pA/pF and TALK-1 KO: 19.41±0.74 pA/pF, P<0.001), and 60 mV (WT: 40.52±1.34 pA/pF and TALK-1 KO: 29.75±1.19 pA/pF, P<0.001).

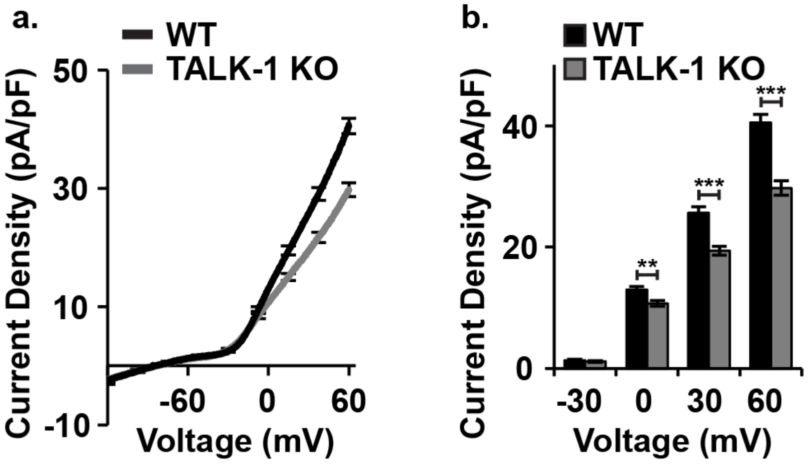

Fig. S1: Knockout of TALK-1 decreases β-cell whole-cell K2P currents. a. Average K2P current density of WT (N=39 cells) and TALK-1 KO (N=31 cells) β-cells and b. average K2P currents in WT (black) and TALK-1 KO (gray) mouse β-cells at selected voltages. Statistical analysis was conducted with unpaired two-tailed t-tests and uncertainty is expressed as SEM (**P<0.01, ***P<0.001).

2. Annexin-V-FITC apoptosis assay for WT and TALK-1 KO β-cells

WT and TALK-1 KO mouse islets were isolated and dispersed into single cells then cultured in islet media for 24 hrs at 37°C, 5% CO_2. The islet media was then replaced with fresh islet media or islet media with cytokines and the islets were cultured an additional 24 hrs at 37°C, 5% CO_2. Apoptotic cells were visualized using an ApoScreen® Annexin V Apoptosis Kit-FITC (SouthernBiotech) as per manufacturer's instructions. Cell nuclei were stained with Hoechst 33342 (Tocris Bioscience) and used to determine total cell number per field. Fluorescence was imaged using a Nikon Eclipse TE2000-U microscope equipped with an epifluorescence illuminator (Sutter Instrument Company), a CCD camera (HQ2; Photometrics, Inc), and Nikon Elements software (Nikon, Inc). Data analysis was performed using the ImageJ Fiji image processing pack.

Because $[Ca^{2+}]_{ER}$ handling is an important factor in apoptotic signaling we investigated the involvement of TALK-1 expression in cytokine-induced β-cell apoptosis (Fig. S2). In the absence of cytokines TALK-1 expression had no effect on β-cell apoptosis (WT nontreated: 22.1±1.1% apoptotic and TALK-1 KO nontreated: 23.8±1.3% apoptotic, Fig. S2). Cytokine exposure led to a significant increase in apoptosis for WT and TALK-1 KO β-cells (WT cytokines: 29.7±1.6% apoptotic, $P<0.05$, and 33.9±1.2% apoptotic, $P<0.001$). However, this increase in apoptosis was indistinguishable for WT and TALK-1 KO β-cells indicating that this rise is not due to changes in TALK-1 expression.

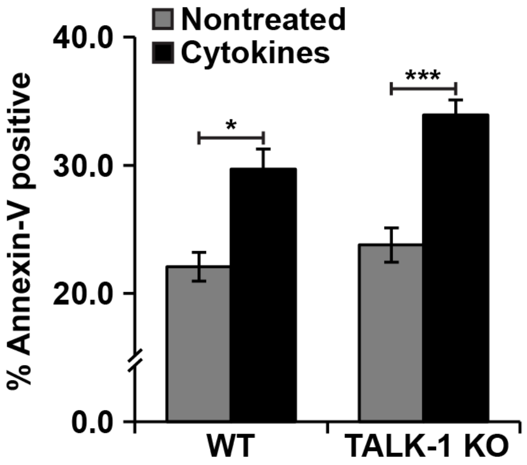

Fig. S2: Cytokine exposure triggers β-cells apoptosis in a TALK-1 independent manner. Percent apoptotic cells calculated as number of annexin-V-FITC positive cells divided by total number of cells (identified by Hoechst 33342 nuclear staining). Average % apoptotic cells for nontreated (gray) and cytokine treated (black) WT (N=3 animals) and TALK-1 KO (N≥3 animals) β-cells. Statistical analysis was conducted with a 1-way ANOVA and uncertainty is expressed as SEM (**P<0.01, ***P<0.001).

3. Quantification of $[Ca^{2+}]_i$ oscillation amplitudes in WT and TALK-1 KO β-cells

Because the amplitude of islet $[Ca^{2+}]_i$ oscillations during 2nd phase GSCI are greatly reduced after exposure to cytokines, the magnitude of this decrease was quantified. Whole islet Fura-2 AM $[Ca^{2+}]_i$ recordings (from Fig. 2) were examined and all islets exhibiting $[Ca^{2+}]_i$ oscillations for a period of at least 5 min were included in this analysis. A 5 min oscillatory period was selected for each recording and the amplitude of $[Ca^{2+}]_i$ oscillations was estimated as $F_{340/380,\ max}$ - $F_{340/380,\ min}$ (Fig. S3a). The $[Ca^{2+}]_i$ oscillation amplitude of nontreated WT and TALK-1 KO islets were indistinguishable (WT: 0.285±0.022 $\Delta F_{340/380}$ and TALK-1 KO: 0.387±0.031 $\Delta F_{340/380}$) After cytokine exposure $[Ca^{2+}]_i$ oscillation amplitude decreased in magnitude ($P<0.001$) similarly in WT and TALK-1 KO islets (WT: 0.119±0.011 $\Delta F_{340/380}$ and TALK-1 KO: 0.130±0.013 $\Delta F_{340/380}$). These results suggest that the cytokine-mediated decrease in islet $[Ca^{2+}]_i$ oscillation amplitude in not related to TALK-1 expression.

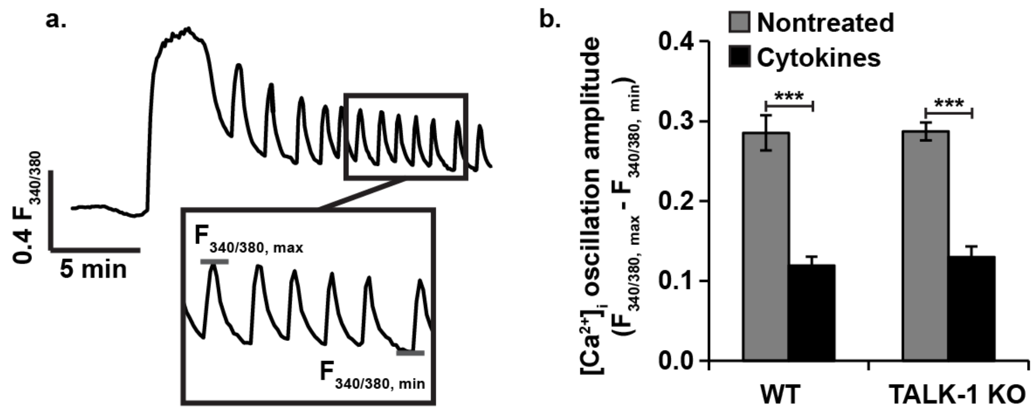

Fig. S3: Cytokine exposure decreased islet $[Ca^{2+}]_i$ oscillation amplitude independently of TALK-1 expression. a. Overview of approach used to quantify average islet $[Ca^{2+}]_i$ oscillation amplitude and b. average $[Ca^{2+}]_i$ oscillation amplitudes for nontreated (gray) and cytokine treated (black) WT (N≥41 islets) and TALK-1 KO (N≥23 islets). Statistical analysis was conducted with a 1-way ANOVA and uncertainty is expressed as SEM (***P<0.001).

4. Unmodified human islet TALK-1 western blots

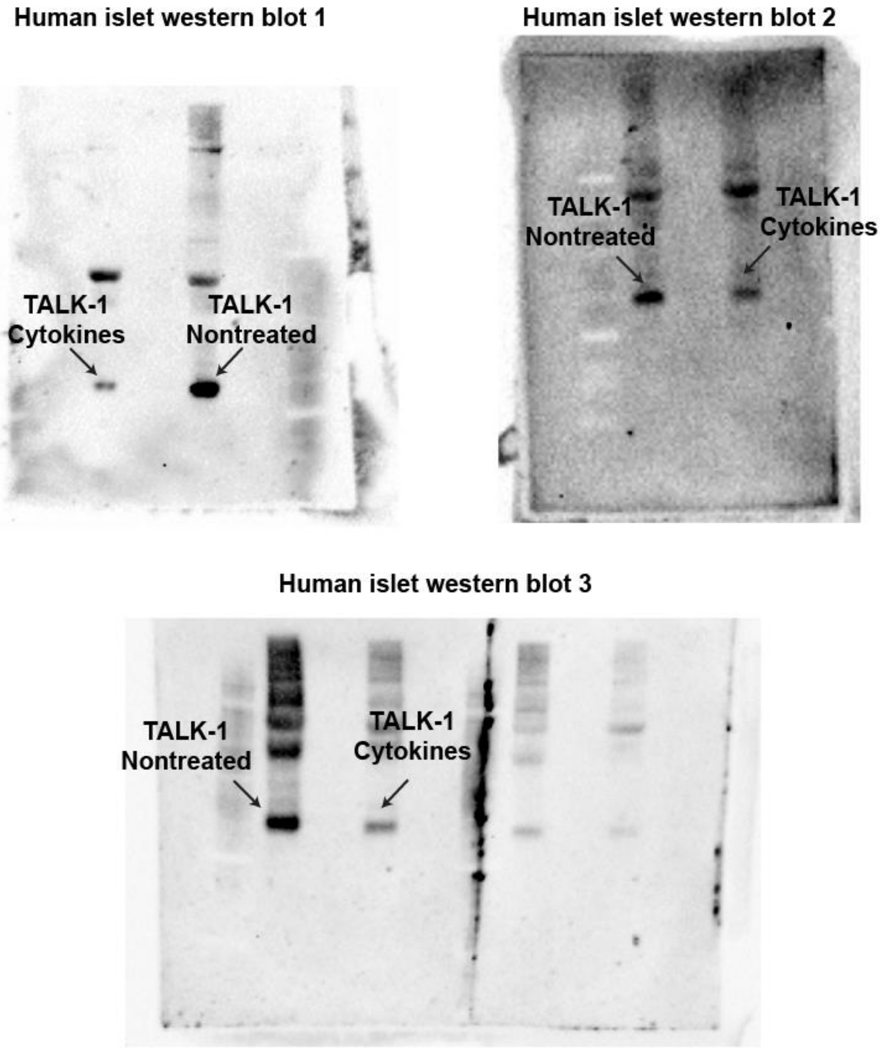

Fig S4: Cytokine exposure reduces TALK-1 protein expression in human islets. Unmodified western blots for TALK-1 protein from human islets quantified in Fig. 1c. For each western blot 40 mg total protein/lane loaded (as calculated by BCA assay).
